# Effects of pre-analytical handling on selected canine hematological parameters evaluated by automatic analyzer

**Published:** 2016-12-15

**Authors:** Labrini Vasileiou Athanasiou, Zoe Polizopoulou, Maria Rafaela Kalafati, George Ntararas, Vasileios Kontos

**Affiliations:** 1 Department of Medicine, Faculty of Veterinary Medicine, University of Thessaly, Karditsa, Greece; 2 Diagnostic Laboratory, Faculty of Veterinary Medicine, Aristotle University of Thessaloniki, Thessaloniki, Greece; 3 National School of Public Health, Department of Veterinary Public Health, Athens, Greece

**Keywords:** Complete blood count, Dog, Hematology, Stability, Temperature

## Abstract

To assess the effects of pre-analytical handling (storage time and temperature) on selected hematological parameters, whole blood samples were collected in EDTA coated tubes from each of 30 clinically normal male adult beagle dogs. Each sample was separated in 2 aliquots, of which one was stored in ambient temperature (25 ˚C) and the other one was refrigerated (2 to 4 ˚C). Complete blood counts were performed in 1, 2.5, 5, 12, 24, 36 and 60 hr post-sampling for each aliquot of every sample using a flow cytometer. Packed cell volume values remained stable in the samples kept in room temperature (RT), whereas a significant increase was noted in the refrigerated ones 24 hr post-sampling. Statistically significant increases in red blood cell counts were noted after 24hr in the samples stored in 2 to 4 ˚C and after 12 hr in those kept in RT. No significant changes were observed in haemoglobin concentration. A significant decrease was evident only 60 hr post-sampling for the white blood cells kept in RT, but not for those kept in 2 to 4 ˚C. Platelet counts significantly decreased after 24 hr in the refrigerated aliquots and after 5 hr in those kept in RT. The results of this study indicate that storage of blood samples for up to 24 hr in 2 to 4 ˚C is associated with the least artifactual changes.

## Introduction

The complete blood count (CBC) is one of the most common routine laboratory tests requested as the first step to diagnose an illness or clinical presentation. With the development of automated hematological analyzers, the CBC has become an easy, quick and reliable test that can give valuable information to clinicians leading to provisional diagnosis and direct further testing.^1^ Even though many companion animal practices are equipped with automated analyzers capable of processing hematological tests in an efficient and timely manner, during the last years, the increasing specialization and centralization of veterinary sample testing into veterinary diagnostic laboratories has dramatically changed the time existing between the blood sampling and the measurement of haematological parameters. This signifies that a high number of blood samples are generally transferred to long distance laboratories for performing the analytical measurement. However, for reliable results, it is essential that blood samples are collected and stored properly and then examined within a specified time frame.^2^^,^^3^ Since storage of blood samples for variable periods of time prior to forwarding to a diagnostic laboratory is common in veterinary practice, it could lead to erroneous results and hinder case differential diagnosis and management.^4^^,^^5^ Furthermore, in contrast to biochemistry^6 ^with hematology analyzers, cells may undergo damages not only by the storage period but also by the specific reagent and/or stain used by each automatic analyser during the analytical process. Consequently, the changes observed may differ according to the analyzer used.^5^ Moreover, there are no published data about the stability of laboratory reared Beagle dogs which may be different from blood of other canine species. Beagles are frequently used as experimental animals for pharmacokinetic studies where alterations in blood parameters could also affect the result of the study.^7^^,^^8^

The purpose of this study was to assess the effects of pre-analytical handling (storage time and temperature) on selected hematological parameters in canine blood samples using a flow cytometer.

## Materials and Methods

 Thirty laboratory-reared, clinically healthy male beagles, approximately two years old were used in this study. The dogs were housed in indoor kennels in social groups of two to three animals, had daily access to an outdoor exercise area and human interaction periods at least twice per day. The dogs were fed a standard commercial dog feed (Hill's Science Diet Canine Adult Maintenance food; Hill’s Pet Nutrition, Topeka, USA) at the quantities suggested by the manufacturer, while water was available for *ad libitum* consumption.

Whole blood samples (10 mL) were collected from the jugular vein by using a 21-gauge needle and syringe placed it into two 5-mL tubes containing 1.6 mg mL^-1 ^EDTA-K3 (Sarstedt AG & Co, Nümbrecht, Germany). One aliquot was stored in room temperature (RT; 25 ˚C) and the other one was refrigerated (2 to 4 ˚C). Complete blood counts were performed in consecutive time intervals (1, 2.5, 5, 12, 24, 36 and 60 hr post-sampling) for each aliquot of every sample using Cell-Dyn 3500 flow cytometer (Abbott Laboratories, Abbott Park, USA). Refrigerated samples were warmed to room temperature prior to analysis. The first three time intervals were selected to test blood stability when it is to be analyzed in clinics or research units equipped with automatic analyzers, while the rest of them when blood is sent to be analyzed in longer distances. In each case the experimental design was considered as a mixed or a split-plot design. The experimental data were subjected to an analysis of variance (ANOVA) in the context of general linear models. The experimental significance level was preset at 5%. The effects of time and storage condition were assessed using ANOVA, followed by Bonferroni’s correction. The statistical software package SPSS (version 20; SPSS Inc., Chicago, USA) was used for data processing.

## Results

**Fig. 1:**
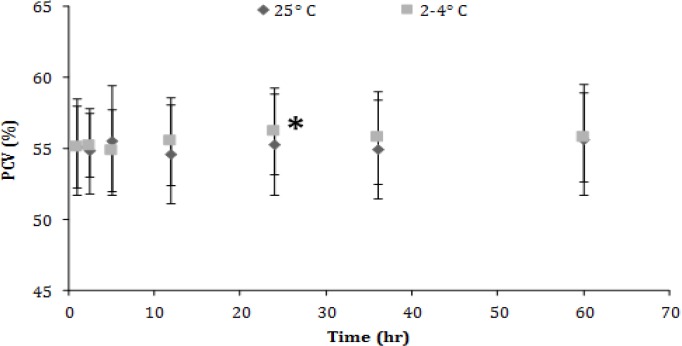
Packed cell volume (PCV) values (mean ± SD) of canine blood samples at different temperatures during 60 hr. Asterisk indicates significant difference compared to the other time points

Packed cell volume (PCV) values remained stable in the samples kept in RT, but a significant increase (*p* < 0.05) was noted in the refrigerated ones after 24 hr of storage (Fig. 1).

**Fig. 2:**
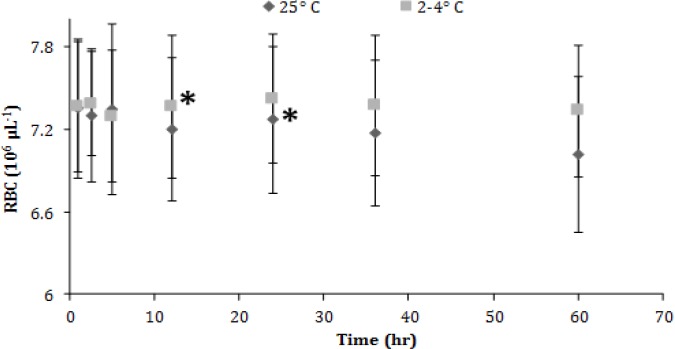
Red blood cell (RBC) count (mean ± SD) of canine blood samples at different temperatures during 60 hr. Asterisks indicate significant difference compared to the other time points

**Fig. 3:**
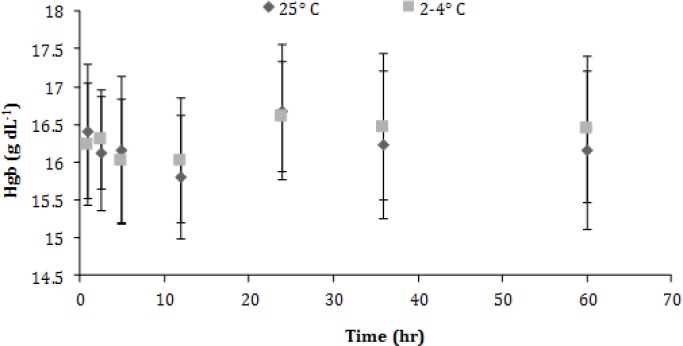
Hemoglobin (Hgb) concentration (mean ± SD) of canine blood samples at different temperatures during 60 hr

Statistically significant increases in red blood cell (RBC) counts were noted after 24 hr in the samples stored under refrigeration and after 12 hr in those kept in ambient temperature (Fig. 2). No significant changes were observed in hemoglobin (Hgb) concentration (Fig. 3).

**Fig. 4:**
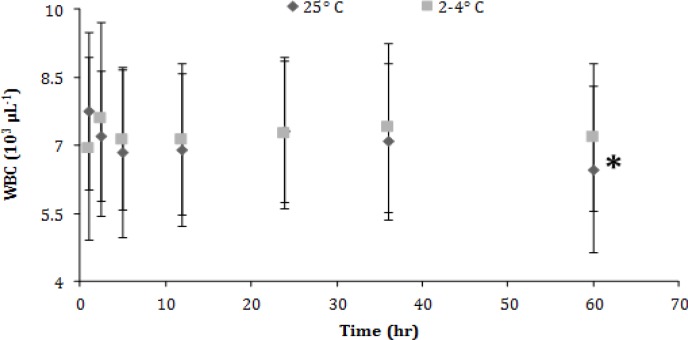
White blood cell (WBC) count (mean ± SD) of canine blood samples at different temperatures during 60 hr. Asterisk indicates significant difference compared to the other time points

**Fig. 5:**
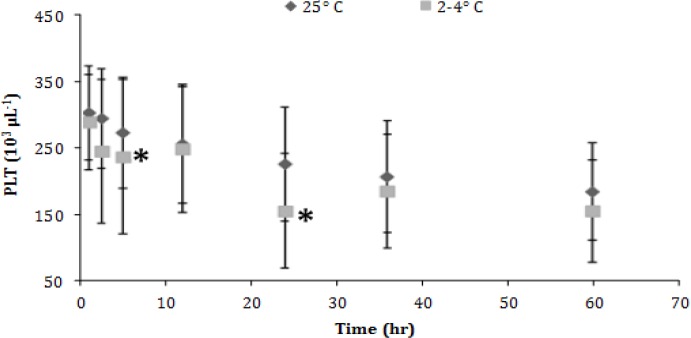
Platelet (PLT) count (mean ± SD) of canine blood samples at different temperatures during 60 hr. Asterisks indicate significant difference compared to the other time points

No significant changes were recorded concerning the white blood cell (WBC) counts in the refrigerated samples, but in those kept in RT a significant decrease (*p *< 0.05) was evident only during the last observation time (60^th^ hr), (Fig. 4). Finally, changes seen in platelet (PLT) counts included significant decreases (*p *< 0.05) after 24 hr in the refrigerated aliquots and after 5 hr in those kept in ambient temperature (Fig. 5).

## Discussion

 Hematological parameters are important to assess the physiological status of patients and monitor pathological changes. The main objective of any laboratory determination is to produce results that are accurate and precise. It is therefore routinely recommended to perform hematologic determinations on blood samples shortly after blood collection, and if not possible, the samples should be refrigerated at 4 ˚C until analysis to minimize artifactual changes.^3^^,^^4^

Delayed analysis can occur when blood is couriered to a reference laboratory or circumstances do not allow for timely analysis. It is well known that handling of blood samples and method of keeping and storage can significantly mislead the results.^9^^-^^13^

Whole blood is usually collected in anticoagulants to prevent them from clotting.^14^ Ethylenediamine tetra-acetic acid (EDTA) is the most common choice for automated blood cell counts.^15^ According to a study, EDTA anti-coagulated blood samples stored over time can possibly lead to changes in erythrocytes morphology and osmotic fragility.^16^ Furthermore, hematology focus on analyze cells, which may undergo autolysis during storage and analytical conditions, because of the reagents and the stains of the specific automatic analyzer. Consequently, the changes observed may differ according to the analyzer used.^5^ The results of the present study were defined statistically, analytically and clinically.

Abbott Cell Dyn 3500 is a fully automated hematological analyzer that combines impedance and laser technologies.^17^ Cell Dyn 3500 is able to implement complete blood cell count and, also, differentiate the WBC counts.^17^^-^^21^ Various domestic animals’ blood can be examined with this analyzer.^17^^,^^22^ Furthermore, validation studies demonstrate that it is an accurate analyzer in veterinary medicine and specifically for canine blood.^23^

To begin with the PCV values’ stability in the samples kept in RT in contrast to the refrigerated samples that increase was expected due to damaged erythrocytes which provoke artifactual increase of the calculated PCV.^1^ These results are partially in line with another study which reports rise in PCV after 12 hr in 24 ˚C and 4 ˚C.^25^ According to the literature, hematocrit presented a significant increase after 12 hr of storage both in RT and refrigerator that continues up to 48 hr and was more obvious in the samples stored at RT.^24^ This increase is the result of the water absorption by erythrocytes.^25^

Previous studies suggest that canine RBC count and Hgb concentration are the most stable variables since alterations have not been mentioned before a minimum time period of two days both in refrigerated and RT storage conditions.^4^^,^^5^^,^^26^ This observation is partially discordant to the results of this study where RBC count increases in 12 hr and 24 hr for the RT and refrigerated samples, respectively. It is worth mentioning that RBC can aggregate during storage, particularly in RT conditions.^27^

White blood cell counts remained nearly constant in both temperatures and only after 60 hr those kept in RT had a considerable drop. The results of similar studies are variable. Compared to other researches, initially WBC remarked a relative increase while after 72 hr the total number of white blood cells decline.^4^ According to another study blood stored in RT had lower WBC counts after 48 hr.^24^ At the same time a third research concludes to the stability of the WBC counts.^5^ The results of the current study, indicate a significant drop in PLT s count after 5 hr of storage in RT and 24 hr in the refrigerator while another study indicates that PLT count can be stable up to four days in RT.^28^ The stability of PLT counts for approximately 48 hr reinforces the result of present study.^29^ Considering the bibliography it is conspicuous that blood cells can aggregate during storage and refrigeration.^30^ Especially, preservation in 4 ˚C tends to provoke more PLT aggregation.^30^

In conclusion, the examined parameters (PCV, Hgb, RBC, WBC, PLT) display acceptable stability up to 24 hr in 4 ˚C according to human and veterinary medicine studies.^29^^,^^31^ However, differences observed in different studies can also be attributed to the different definitions of stability and different methods of determination. Apart from the analytical and statistical differences, it is important for the practitioners to know if the effect of storage and temperature can change the diagnosis. In this context, the sample was considered stable when the result was within reference range and did not change diagnosis and therapeutic interventions. From this point of view, blood was stable for all parameters 12 hr after sampling when kept in the refrigerator.

Stability seems to be different for different hematological parameters depending on the storage temperature and also the specific analyzer.^32^ The results of this study indicate that storage of blood samples for up to 24 hr in 2 to 4 ˚C is associated with the least artifactual changes.
